# Combining AdipoRon with Paclitaxel Unveils Synergistic Potential in Non-Small Cell Lung Cancer Cells via AMPK-ERK1/2 Signaling

**DOI:** 10.3390/cells14080602

**Published:** 2025-04-16

**Authors:** Sanober Kafeel, Giuseppina Palmiero, Alessia Salzillo, Angela Ragone, Silvio Naviglio, Luigi Sapio

**Affiliations:** 1 Department of Precision Medicine, University of Campania “Luigi Vanvitelli”, 80138 Naples, Italy; sanober.kafeel@unicampania.it (S.K.); giuseppina.palmiero@unicampania.it (G.P.); alessia.salzillo@unicampania.it (A.S.); or angela.ragone@mpi-dortmund.mpg.de (A.R.); luigi.sapio@unicampania.it (L.S.); 2 Department of Mechanistic Cell Biology, Max Plank Institute of Molecular Physiology, 44227 Dortmund, Germany

**Keywords:** NSCLC, AdipoRon, Paclitaxel, apoptosis, AMPK, ERK1/2

## Abstract

As part of chemotherapy regimens, Paclitaxel improves the overall survival of many non-small cell lung cancer (NSCLC) patients. However, the development of drug resistance and adverse events limits its clinical usage, reinforcing the need for further advancements in NSCLC therapeutics. We recently recognized the adiponectin receptor agonist AdipoRon as a promising anticancer compound in NSCLC. Consequently, this study aimed to evaluate the therapeutic potential of combining AdipoRon with Paclitaxel (Combo) in NSCLC cells. With respect to individual treatments, Combo triggered a stronger inhibition of both cell growth and clonogenic potential, as well as a greater induction of cell death. The Combo-mediated cytotoxicity was also corroborated by cleavage of poly-ADP ribose polymerase (PARP) and caspase-3 apoptotic markers. Notably, AMP-activated protein kinase (AMPK) emerged as a critical sensor in Combo efficacy, as its inhibition by Compound-C unveiled a significant rescue in cell growth. Although Combo caused a gradual downregulation of extracellular signal-regulated kinase 1/2 (ERK1/2), the hindrance in the upstream cascade by PD98059 partially counteracted the Combo outcomes. In conclusion, our findings designate AdipoRon as an effective candidate in Paclitaxel-based therapy. Nevertheless, future studies aimed at exploring the Combo aptitude in overcoming the Paclitaxel-related restraints need to be investigated in NSCLC.

## 1. Introduction

NSCLC is a heterogeneous malignant disorder with various genetic and molecular features that accounts for approximately 85% of all lung cancer cases [1]. Despite the latest advancements achieved in both targeted and immune therapies, the prognosis of NSCLC patients remains dismal enough to be considered the leading cause of cancer-related death worldwide [2]. Chemotherapy still represents a cornerstone in NSCLC care, especially in managing patients who are proceeding to the advanced stages [3]. Among chemotherapeutic interventions, Paclitaxel is widely accepted as a reference regimen for advanced, recurrent, or metastatic NSCLCs. Approved by the Food and Drug Administration (FDA) for the treatment of NSCLC and many other tumors, Paclitaxel is a taxane derived from the bark of the Pacific yew tree, *Taxus brevifolia* [4]. The administration of this drug at optimum doses for a prolonged time has been hampered by the prevalence of serious side effects, such as neutropenia, peripheral neuropathy, skin reactions, allergic reactions, bradycardia, and hypotension, as well as the acquisition of tumor resistance [5,6]. As a microtubule-stabilizing agent, Paclitaxel prevents the normal disassembly during cell division, triggering apoptosis, autophagy, and mitotic catastrophe [7]. Paclitaxel incorporation into combination regimens, particularly with platinum-based drugs, has further enhanced its anticancer efficacy [8]. However, resistance mechanisms and dose-limiting toxicities continue to challenge clinical outcomes. Therefore, considerable attention has been given to the identification of new therapeutic agents capable of providing a synergistic effect when combined with Paclitaxel.

As a final deliverable of chemical library screening, AdipoRon was among the first synthetic compounds to bind the adiponectin receptors and activate the stress-responsive AMP-activated protein kinase (AMPK) [9]. Mimicking the insulin-sensitizing and anti-inflammatory features of adiponectin, this compound was initially proposed as a pharmacological intervention for metabolic syndrome [10,11]. Meanwhile, AdipoRon is forcefully emerging as a promising anticancer compound in various types of tumors [12]. Besides the canonical AMPK, modulation of different pathways has been reported in AdipoRon-treated cells, such as mitogen-activated protein kinases (MAPK), mammalian target of rapamycin (mTOR), nuclear factor kappa-light-chain-enhancer of activated B cells (NF-κB), receptor-interacting protein (RIP) kinase family members (RIPKs), and unc-51-like autophagy-activating kinase 1 (ULK1) [13,14,15,16,17]. More recently, a potential AdipoRon involvement in cancer metabolic reprogramming has been assumed in pancreatic and thyroid malignancies [15,18]. It has also been postulated that AdipoRon may potentiate the effects of existing chemotherapeutics by targeting essential metabolic pathways for cancer cell survival and proliferation [19]. Employing AdipoRon as a potential partner in chemotherapy-based therapy is quite new in pharmacology. In this respect, we previously tested the AdipoRon outcome in combination with Gemcitabine in pancreatic ductal adenocarcinoma (PDAC) [20]. Simultaneous administration of AdipoRon and Gemcitabine revealed a stronger action in contrasting PDAC cell growth than single administration. Remarkably, the combination was also persistent in limiting the progression of Gemcitabine-resistant cells. We endorsed the therapeutic candidacy of AdipoRon even in NSCLC, demonstrating a prominent cell growth inhibition along with an adaptive glycolytic dependence lately [21]. However, its synergistic potential in combination with cytotoxic agents remains largely unexplored in NSCLC therapeutics.

Combination therapies have proven to be more effective than monotherapy in treating cancers [22]. Besides potentiating the therapeutic efficacy of each agent, this pharmacological approach enables the use of reduced doses, thus reducing the chances of development of both drug resistance and adverse reactions. As research continues to unravel the complexities of NSCLC biology, exploring the interplay between Paclitaxel and AdipoRon may provide valuable insights for more effective therapeutic strategies in NSCLC. In this study, we aimed to investigate the synergistic potential of AdipoRon and Paclitaxel in minimal doses to enhance their individual antitumor efficacy in NSCLC cells.

## 2. Materials and Methods

### 2.1. Cell Culture and Drug Preparation

American Type Culture Collection (ATCC, Manassas, VA, USA)-derived A549 (CCL-185) and NCI-H1299 (CRL-5803) human NSCLC cell lines were handled according to the manufacturer’s guidelines. Dulbecco’s Modified Eagle’s Medium (ECM0728L; EuroClone, Pero, Italy) and RPMI-1640 Medium (ECB9006L; EuroClone, Pero, Italy), supplemented with 10% Fetal Bovine Serum (ECS5000L; EuroClone, Pero, Italy), 1% Penicillin–Streptomycin Solution 100X (ECB3001D; EuroClone, Pero, Italy), and 1% L- Glutamine 100X (ECB3000D; EuroClone, Pero, Italy), were employed to culture A549 and H1299 cells, respectively. Cells were maintained in controlled sterile conditions, typically at 37 °C with 5% CO_2_ and a 95% humidified atmosphere. For each experimental design, cells were seeded in a fixed number and then allowed to settle freely in order to attain 20–30% confluence within 24 h. On the subsequent day, culture media were replaced with fresh media along with supplementation of different drugs as demanded by each experimental plan. The following drugs and stock solutions were employed in this study: AdipoRon (10 mg/mL), Paclitaxel (10 μM), Compound-C (10 mM), and PD98059 (10 mM). Since drug reconstitution required dimethyl sulfoxide (DMSO), the same diluent amount was included in the investigation setting as a negative control.

### 2.2. Chemical Reagents

AdipoRon (#57583; Cell Signaling Technology, Danvers, MA, USA), Paclitaxel (#9807; Cell Signaling Technology, Danvers, MA, USA), Compound-C (EMD Millipore Corp., Burlington, MA, USA), PD98059 (P215; Sigma-Aldrich, St. Louis, MO, USA), Ethanol absolute an- hydrous (308603; Carlo Erba, Cornaredo, Italy), MTT solution (M2128; Sigma-Aldrich, St. Louis, MO, USA), Tween 20 (TC287; HIMEDIA, Mumbai, India), Phosphate-buffer solution (PBS) (ECB4004L; EuroClone, Pero, Italy), Propidium Iodide (#P4864; Sigma-Aldrich, St. Louis, MO, USA), RNase A (R5503; Sigma-Aldrich, St. Louis, MO, USA), RIPA buffer (R0278; Sigma-Aldrich, St. Louis, MO, USA), Protease/Phosphatase Inhibitor Cocktail (#5872; Cell Signaling Technology, Danvers, MA, USA), Trypsin-EDTA 1X in PBS (ECB3052D; EuroClone, Pero, Italy), 2X Laemmli (S3401; Sigma-Aldrich, St. Louis, MO, USA), Ponceau staining solution (A2935; PanReac AppliChem, Castellar del Vallès, Spain), Crystal violet (#C0775; Sigma-Aldrich, St. Louis, MO, USA), DMSO (A3672; PanReac AppliChem), Non-fat dry milk (A0530; PanReac AppliChem, Castellar del Vallès, Spain), Amersham Protran Premium 0.45 μm nitrocellulose membrane (10600008; Cytiva, Marlborough, MA, USA), and Excellent Chemiluminescent Substrate Kit (E-IR-R301; Elabsciences, Houston, TX, USA).

### 2.3. Cell Proliferation Assay

Cell proliferation was assessed by cell counting with an optical microscope. A549 and H1299 cells were seeded at a density of 6 × 10^4^ in a 6-well plate and treated differentially as required by each experimental plan. At predetermined time points, cells were detached using Trypsin-EDTA 1X (ECB3052D; EuroClone, Pero, Italy), and counted with either Hirschmann™ Counting Chamber (8110101; Fischer Scientific, Waltham, MA, USA) or CytoSMART (6749; Corning, NY, USA). At least three biological replicates were carried out for each experiment and displayed in figures as means ± standard deviation.

### 2.4. PI Exclusion Assay

The propidium iodide (PI) exclusion assay was carried out to discriminate the proportion of live and dead cells in response to drug stimulations. As a small fluorescent molecule, PI can bind nuclear DNA only when the permeability of cell membranes is severely compromised, such as in the culminating step of cell death [23]. Briefly, a mixture of both floating and detached cells was spun down at 1200 rpm for 5 min. The cell pellet was washed once with PBS and resuspended in PBS containing PI (4 μg/mL). PI-stained cells were immediately subject to estimation by FACSCalibur™ (BD Biosciences, Franklin Lakes, NJ, USA), recording at least 20,000 events for each sample. The percentage of dead and live cells was recorded in each condition and used to analyze the cell death induction by column chart.

### 2.5. Cell Cycle Analysis

The cell population was differentiated into various phases of the cell cycle by PI staining. Specifically, cells at a density of 6 × 10^4^ were seeded and treated in a predetermined manner using 6-well plates. After the spin cycle (at 1200 rpm for 5 min), the pellet was washed with PBS before being dissolved in 300 µL PBS plus 700 µL of drop-by-drop chilled absolute ethanol. Preservation of fixed samples was performed at −20 °C until estimation. Once ready for the analysis, the samples centrifugation was performed at 1200 rpm for 5 min before being resuspended in a mixture of PI (15 μg/mL) and RNase A (10 μg/mL) in PBS. After 10 min of incubation in the dark, 20,000 events/sample were recorded by FACSCelesta™ (BD Biosciences, Franklin Lakes, NJ, USA). Gating strategy was applied to establish the relative amount for each of the following cell cycle phases: subG1 (<2n), G0/G1 (2n), and G2/M (4n). Replicative phase (2n–4n) was mathematically derived through the formula S=100−(G0/G1+G2/M+subG1). At least three biological replicates have been carried out for the assessment of cell cycle analysis.

### 2.6. Colony Forming Assay

The colony formation assay (CFA), also known as the clonogenic assay, allows the investigation of the stemness by measuring the cell’s ability to proliferate and differentiate into colonies. A549 and H1299 cells were seeded at a very low density (1.5 × 10^3^/well) in 6-well plates and treated with AdipoRon, Paclitaxel, and Combo on the subsequent day. Colonies were stopped after ten days and stained with a 1% aqueous solution of crystal violet for 10 min. Washing with distilled water was performed two times to remove the unbound traces of the dye. Lastly, colonies were naturally dried before being captured by a digital camera. Quantification analysis was accomplished in a two-stage procedure, involving solubilization of the stained colonies in 10% acetic acid and measurement of the optical density (OD) at 590 nm by Infinite 200 PRO Microplate Reader (Tecan Life Sciences, Männedorf, Switzerland). Colonies experiments were performed in three biological replicates before formulating statistical considerations.

### 2.7. Cell Viability

Cell viability was measured using the MTT assay (3-(4,5-dimethylthiazol-2-yl)-2,5 diphenyltetrazolium bromide). Briefly, 1.8 × 10^3^ cells were cultured in 96-well plates and treated with 200 μL of medium containing or not (Control) three different concentrations of AdipoRon and Paclitaxel, as well as all their possible combinations. After 48 h of treatment, 10 μL of MTT solution (1 mg/mL) was added to each well to allow the formation of MTT formazan crystals. After three hours of incubation at 37 °C, the medium was removed, and crystals were solubilized in 100 μL of Isopropanol-HCl 0.04 N using horizontal shaking. The absorbance was recorded at 570 nm using an Infinite 200 PRO Microplate Reader (Tecan Life Sciences, Männedorf, Switzerland).

### 2.8. Multi-Drug Synergy Analysis

Cell viability results were used to calculate the inhibition rate in response to AdipoRon, Paclitaxel, and Combo for both A549 and H1299 cell lines. Synergistic drug interaction was evaluated in a 4 × 4 matrix by the SynergyFinder web application (version 3.0). Based on multi-dose experimental analysis, the software provides a synergy scoring method (called Bliss/Loewe consensus) that combines multiple reference models (Bliss, Loewe, and HSA). The synergy score also outlines the types of drug–drug interaction, where a value lower than −10 indicates antagonistic effects, from −10 to 10 additive, and higher than 10 for synergistic ones [24].

### 2.9. Protein Extraction and Quantification

Sample protein extracts were prepared by seeding 4 × 10^5^ cells into 100 mm Petri dishes. After incubation with either single or combined drugs, cells were collected by scraping and spun down at 1200 rpm for 5 min. The cell pellets were immediately frozen at −80 °C until protein extraction and quantification. Following thawing, the cells were lysed using RIPA buffer containing a Protease and Phosphatase Inhibitor Cocktail. The lysis process involved three cycles of vortexing with 10 min intervals, interspersed with 4 °C centrifugation at 12,000 rpm for 20 min. The protein concentration in the cell lysate supernatant was then measured by Bradford assay (A6932,0500; PanReac AppliChem ITW Reagents, Castellar del Vallès, Spain), using Bovine Serum Albumin (BSA) (A6588; PanReac AppliChem ITW Reagents, Castellar del Vallès, Spain) as a calibration standard. The absorbance was recorded at 595 nm using a UV/VIS Spectrophotometer V-550 (JASCO, Mary’s Court Easton, MD, USA). All procedures were carried out at 4 °C to protect the sample integrity.

### 2.10. Immunoblotting

Immunoblotting samples were prepared by combining the total protein extract with 2× Laemmli buffer. After that, samples were first boiled at 95 °C for 6 min and then centrifuged at 6500 rpm for 5 min. Equal protein concentrations (20–30 μg) were loaded for each sample. Lysates were resolved by sodium dodecyl sulfate–polyacrylamide gel electrophoresis (SDS-PAGE) on 8–14% gels at 135 V. Proteins were then transferred to nitrocellulose membrane using wet transfer at 100 V for 90 min in a Mini Trans-Blot system (1703930; Bio-Rad Laboratories; Hercules, CA, USA). Membrane integrity and transfer efficiency were verified by Ponceau S staining. Following three washes with tris-buffered saline containing 0.05% Tween^®^ 20 (TBS-T), the membrane was blocked with 5% non-fat dry milk for 1 h. The membrane was then incubated overnight at 4 °C with the appropriate primary antibodies, followed by washing with TBS-T and incubation for 1 h with anti-species HRP-conjugated secondary antibody. The following antibodies were employed for Western blotting: Cyclin A (H-432) (sc-751) was purchased from Santa Cruz Biotechnology; Cleaved Caspase-3 (Asp175) (5A1E) (#9664), PARP (#9542), Vinculin (#13901), α-Tubulin (DM1A) (#3873), AMPKα (#2532), phosphor-AMPKα (#2535), p44/42 MAPK (#9102), phospho-p44/42 MAPK (#9101), p21 Waf1/Cip1 (12D1) (#2947), goat anti-rabbit IgG HRP-linked (#7074), and goat anti-mouse IgG HRP-linked (#7076) were obtained from Cell Signaling Technology (Danvers, MA, USA). All the stated antibodies were diluted 1:1000 in T-TBS solution containing 5% *w*/*v* BSA, except for Cyclin A, α-Tubulin, goat anti-rabbit IgG HRP-linked, and goat anti-mouse IgG HRP-linked, which were resuspended in T-TBS supplemented with 5% non-fat dry milk. Chemiluminescence was detected using an Excellent Chemiluminescent Substrate Kit (E-IR-R301; Elabsciences, Houston, TX, USA), captured using a ChemiDoc XRS+ System (Bio-Rad Laboratories, Hercules, CA, USA), and quantified using ImageJ software (Version 1.52a).

### 2.11. Statistical Analysis

Data were reported as mean ± standard deviation of three biological replicates for each experiment. For assessment of statistical significance, Brown–Forsythe and Welch ANOVA test or Welch’s *t*-test was used, depending on the number of experimental groups. All statistical tests were performed on GraphPad Prism software (Version 8.0.2) using the following thresholds of significance: * *p* < 0.05, ** *p* < 0.01, *** *p* < 0.001, and **** *p* < 0.0001.

## 3. Results

### 3.1. Combination of AdipoRon Plus Paclitaxel Enhances Cell Growth Inhibition and Impairs Clonogenic Potential in NSCLC Cells

We previously demonstrated that AdipoRon exerts antiproliferative features in NSCLC cells [21]. On the other side, Paclitaxel still remains one of the widely used chemotherapy drugs for the treatment of NSCLC [25]. Hence, we speculated on what the combined effects of these two drugs could be on the therapeutics of NSCLC. To pursue our aim, A549 and H1299 cells were initially treated with 10 µg/mL AdipoRon and 4 nM Paclitaxel for 48 h, either individually or in combination. This choice reflected the need to use submaximal concentrations for both compounds. As a result of previous dose–effect curves [21], 10 µg/mL was absolutely in compliance with other evidence in which AdipoRon acts as an antiproliferative agent [13,15,17,20,26]. Analogously, 4 nM arose for the low impact among the Paclitaxel dosages tested in the past [27].

**Figure 1:**
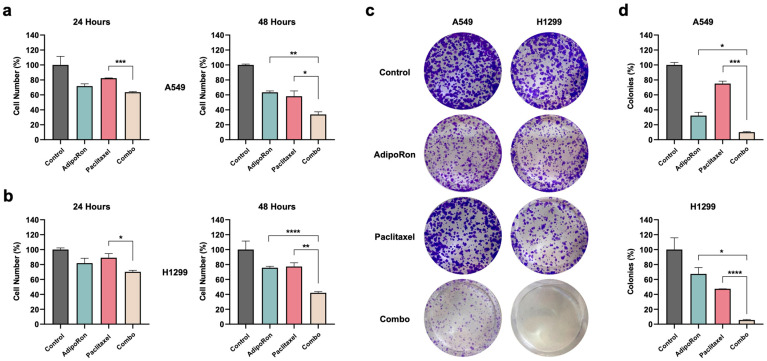
Evaluation of the AdipoRon, Paclitaxel, and Combo-mediated effects on cell growth and colony formation in NSCLC cell lines. (**a**) A549 and (**b**) H1299 cells were treated or not (Control) with 10 µg/mL of AdipoRon, 4 nM of Paclitaxel, and Combo for 24 and 48 h. Cell growth was estimated by direct counting and depicted on the graph as a percentage of the Control. CFA was assessed after nine days of treatment without (Control) or with 10 µg/mL of AdipoRon, 2 nM of Paclitaxel, and their combination in A549. A different dosage of both AdipoRon (5 µg/mL) and Paclitaxel (4 nM) was employed in H1299. Representative Crystal violet-stained colonies of A549 and H1299 are shown in panel (**c**), while quantification analysis from multiple independent experiments is stated in panel (**d**). All the reported tests were replicated at least three times. * *p* < 0.05, ** *p* < 0.01, *** *p* < 0.001, **** *p* < 0.0001 by Brown–Forsythe and Welch ANOVA tests.

Remarkably, Combo appeared as the most effective treatment in inhibiting NSCLC cell growth, reaching a value higher than 66% in A549 and 58% in H1299 at 48 h (Figure 1a,b). Focusing on the longest time point, Combo enhanced the single outcome of AdipoRon and Paclitaxel by approximately 29.6% and 24.3% in A549, respectively (Figure 1a). An even stronger trend was depicted in H1299, where Combo boosted the inhibition rate of AdipoRon and Paclitaxel by a further 33.5% and 35.1%, respectively (Figure 1b). Significant improvement was also detected against Paclitaxel at 24 h, as supported by 36.5% and 29.9% inhibition recorded with Combo in A549 and H1299, respectively (Figure 1a,b).

Considering the clonogenic assay as a suitable test for evaluating self-renewal and differentiation features of cancer cells, we later examined the stemness of the NSCLC cells against single and combined treatments with AdipoRon and Paclitaxel [28]. Impressively, Combo nearly eradicated the formation of new colonies in NSCLC cells (Figure 1c,d). Clonogenic potential of Combo-treated A549 cells dropped to around 90.0% with respect to the untreated counterpart, improving AdipoRon and Paclitaxel performances by 22% and 64.9%, respectively (Figure 1d). Analogously, Combo limited the colony formation of H1299 cells by an additional 61.8% compared to AdipoRon and 41.8% against Paclitaxel alone (Figure 1c,d).

Altogether, these findings indicate that AdipoRon and Paclitaxel work efficiently together in limiting the cell proliferation and colony formation of NSCLC cells.

### 3.2. Combination of AdipoRon Plus Paclitaxel Displays Synergic Interplay in NSCLC Cells

**Figure 2:**
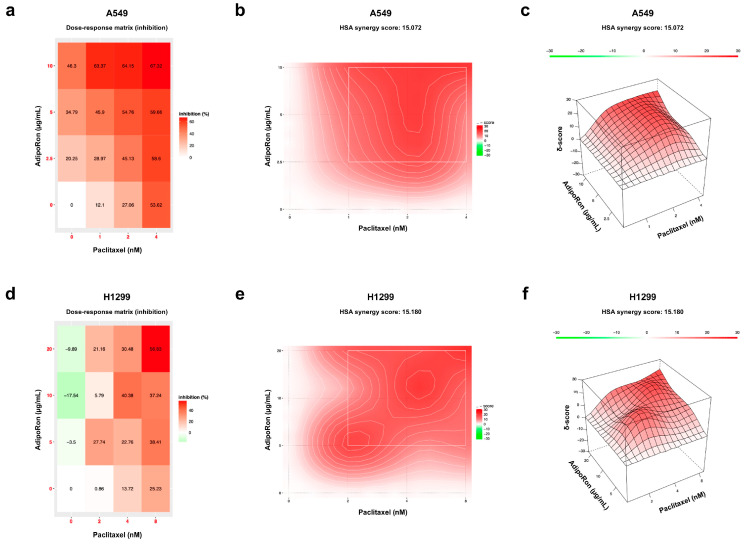
Estimation of the synergistic interplay between AdipoRon and Paclitaxel in NSCLC cell lines. (**a**) Three doses of AdipoRon (2.5, 5, and 10 µg/mL) and Paclitaxel (1, 2, and 4 nM) were employed to develop a dose–response matrix in A549 cells. Highest Single Agent 2D (**b**) and 3D plot (**c**) indicating the synergy score for A549. (**d**) Three doses of AdipoRon (5, 10, and 20 µg/mL) and Paclitaxel (2, 4, and 8 nM) were used to form a dose–response matrix in H1299 cells. Highest Single Agent 2D (**e**) and 3D plots (**f**) indicating the synergy score for H1299. Dose–response matrices display the percentage of inhibition relative to the untreated counterpart.

With the advances in high-throughput drug screening technologies, an increasing number of drug combinations have been tested in cancer models [29,30]. The challenge is the recognition of synergistic interactions that could result in improved therapeutic responses. Due to the complexity of distinguishing additive from synergistic effects, mathematical models can help in defining the relative drug–drug interaction [31]. In view of the promising results achieved by Combo treatment, we hypothesized that a pharmacokinetic relationship could exist between AdipoRon and Paclitaxel in NSCLC cells. Therefore, we employed the SynergyFinder software to assess the synergy score for the proposed combination. Applying a threshold to the most synergistic area (MSA), which represents the most effective 3-by-3 dose-window in a dose–response matrix, the synergy score discriminates among antagonistic (lower than 10), additive (between −10 to 10), and synergistic (higher than 10) interactions [24]. Therefore, MTT-derived results were used to develop a 4 × 4 matrix comprising three distinct AdipoRon and Paclitaxel dosages, as well as all their possible combinations (Tables S1 and S2, Figure 2a,d). Basically, starting from the selected working concentrations (10 µg/mL AdipoRon plus 4 nM of Paclitaxel), we included two additional dosages for both drugs using a constant dilution rate. Notably, the Highest Single Agent (HSA) model depicted a synergy score of 15.07 in A549 (Figure 2b,c) and 15.18 in H1299 (Figure 2e,f), thus suggesting a robust synergistic interaction between AdipoRon and Paclitaxel.

### 3.3. Combination of AdipoRon Plus Paclitaxel Affects Cell Cycle Progression in NSCLC Cells

**Figure 3:**
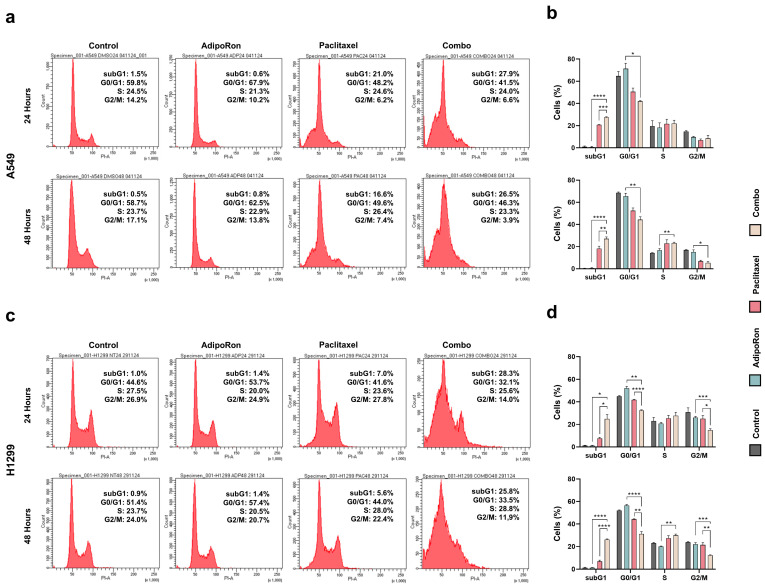
Assessment of the AdipoRon, Paclitaxel, and Combo-mediated outcomes on cell cycle distribution in NSCLC cell lines. A549 and H1299 cells were treated without (Control) or with AdipoRon 10 µg/mL, Paclitaxel 4 nM, and their combination. Panels (**a**,**c**) show representative histogram plots achieved in A549 and H1299 after individual and combined treatments at 24 and 48 h, respectively. Statistical analysis of at least three independent experiments is reported in (**b**) for A549 and (**d**) for H1299. Data are represented in percentages as mean ± standard deviation. * *p* < 0.05, ** *p* < 0.01, *** *p* < 0.001, **** *p* < 0.0001 by Brown–Forsythe and Welch ANOVA tests.

To fully understand the Combo-mediated outcome in NSCLC cells, DNA content was later measured by PI staining with the purpose of evaluating the relative distribution across the different cell cycle phases. Therefore, A549 and H1299 cells were medicated with individual and combined treatment of 10 µg/mL AdipoRon and 4 nM Paclitaxel for up to 48 h. As we previously reported [21], AdipoRon caused an increment in the G0/G1 phase at the expense of the S and G2/M phases in both A549 and H1299 cells, most prominently at 24 h (Figure 3a–d). The appearance of subG1 was the primary Paclitaxel-induced modulation affecting NSCLC cells instead (Figure 3a,c). However, while the subG1 abundance remained almost unchanged over time, A549 and H1299 exhibited a distinctive responsiveness to Paclitaxel. Specifically, the percentage of cells having a DNA content corresponding to subG1 phase was three times higher in Paclitaxel-treated A549 cells than in H1299, regardless of the time dependency (Figure 3a,c). We also observed a reduction in both G0/G1 and G2/M phases as a consequence of Paclitaxel administration in A549 (Figure 3a,b). Corresponding Paclitaxel-mediated G0/G1 changes were also detected at 48 h in H1299 (Figure 3c,d). Considering the Combo outcome, significant variations reshaped the cell cycle profile in NSCLC cells. In particular, compared to Paclitaxel alone, Combo-related subG1 shifted from 20.8% to 27.5% at 24 h in A549 cells (Figure 3b), while the accumulation was even stronger in H1299 cells (from 7.6% to 24.9%) (Figure 3d). No time dependency of the pharmacokinetics was observed for the subG1 occurrence, even in Combo. With respect to AdipoRon treatments, Combo reduced the percentage of both dividing and non-dividing cells (Figure 3a–d). At 48 h, for instance, G0/G1 dropped to 44.3% in A549 and to 31.2% in H1299 cells, with a net decrease of 21.2% and 24.5% compared to AdipoRon alone (Figure 3b,d).

**Figure 4:**
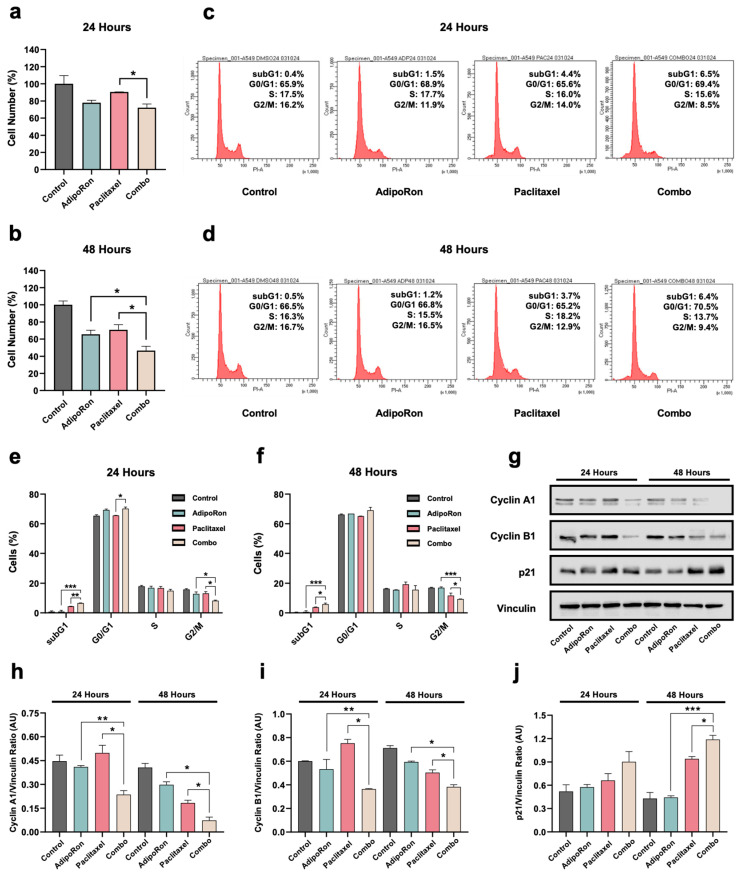
Examination of the low dosages of AdipoRon plus Paclitaxel in A549 cells. Growth response achieved in the presence of 5 µg/mL AdipoRon, 2 nM Paclitaxel, and Combo at 24 (**a**) and 48 (**b**) hours. Representative cell cycle profile obtained in A549 at 24 (**c**) and 48 (**d**) hours. Statistical analysis of multiple cell cycle experiments carried out at 24 (**e**) and 48 (**f**) hours. Typical Western blotting images of Cyclin A1, Cyclin B1, and p21 are reported in panel (**g**) along with their relative quantification (**h**–**j**). Vinculin was used as an internal loading control. All data are displayed in percentages as mean ± standard deviation. For multigroup comparisons, the significance is indicated * *p* < 0.05, ** *p* < 0.01, *** *p* < 0.001 by Brown–Forsythe and Welch ANOVA tests.

To better characterize the Combo outcome on cell cycle progression, we subsequently tested very low dosages of both AdipoRon (5 μg/mL) and Paclitaxel (2 nM) in an effort to minimize the subG1 occurrence in NSCLC cells. Once the efficacy of Combo was validated even at low doses (Figure 4a,b and Figure S2a), we inspected their implications on the cell cycle profile. As anticipated, we obtained negligible subG1 appearance for both Combo (6%) and Paclitaxel (4%) in A549 cells (Figure 4e,f). Minimal changes were also detected in response to AdipoRon and Paclitaxel alone in the other cell cycle stages (Figure 4c,d). On the contrary, we noticed a significant G2/M decline (from 13.1% to 9.4%) in the presence of both drugs at 24 h (Figure 4c,e). Combo-mediated cell cycle fluctuations were further corroborated by the expression levels of Cyclin A1, Cyclin B1, and Cyclin Dependent Kinase Inhibitor p21 Waf1/Cip1 (Figure 4g–j). A sudden massive increase in subG1 was evident in H1299 even by combining low dosage of both AdipoRon and Paclitaxel, thus recognizing this occurrence as a predominant distinctive outcome of Combo in these cells (Figure S1b,c).

Taken together, these data demonstrate that combining AdipoRon with Paclitaxel enhances Paclitaxel-mediated subG1 induction in NSCLC cells. However, substantial changes can also occur in other cell cycle stages when these two drugs are concurrently administered.

### 3.4. Combination of AdipoRon Plus Paclitaxel Improves Chemotherapy-Induced Apoptosis in NSCLC Cells

**Figure 5:**
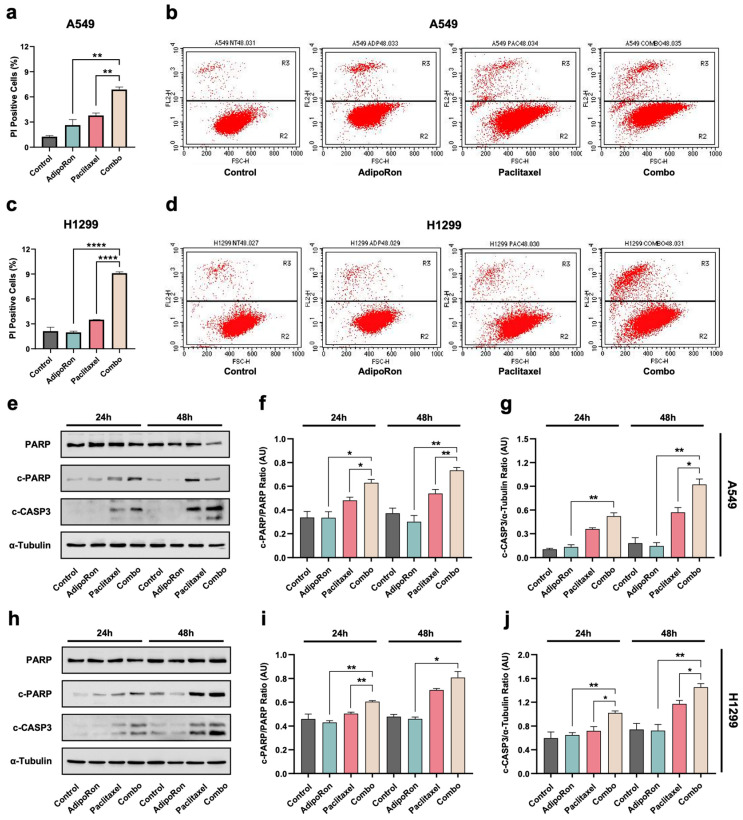
Investigation of the AdipoRon, Paclitaxel, and Combo-induced cell death in NSCLC cell lines. A549 and H1299 were treated without (Control) or with 10 µg/mL AdipoRon, 4 nM Paclitaxel, and Combo for 48 h before undergoing PI exclusion assay. Panels (**a**,**c**) show the analytic results of multiple experiments carried out in A549 and H1299 cells, respectively. Typical dot plots obtained in A549 (**b**) and H1299 (**d**) cells. Apoptotic markers were assessed in A549 and H1299 cells cultured without (Control) or with 10 µg/mL AdipoRon, 4 nM Paclitaxel, and Combo for both 24 and 48 h. Representative Western blotting images are shown in panel (**e**) for A549 and (**h**) for H1299. Cleaved on total PARP ratio (c-PARP/PARP) detected in A549 (**f**) and H1299 (**i**) cells. Cleaved caspase 3 on α-Tubulin ratio (c-CASP3/α-Tubulin) obtained in A549 (**g**) and H1299 (**j**). * *p* < 0.05, ** *p* < 0,01, **** *p* < 0.0001 by Brown–Forsythe and Welch ANOVA tests.

Since the subG1 population gathered the hypodiploid proportion of cells having lost some of their DNA content, we further tested the assumption that combining AdipoRon with Paclitaxel could enhance the chemotherapy-mediated toxicity. To corroborate this hypothesis, we examine the impact of individual and combined treatment with 10 µg/mL AdipoRon and 4 nM Paclitaxel on cell death. The PI exclusion assay was used to monitor the plasma membrane integrity in response to our stimuli at 48 h. Interestingly, Combo exhibited an intensification of dead cells, amounting to 7% in A549 (Figure 5a,b) and 9% in H1299 cells (Figure 5c,d). Non-living cells increased to 7.1% and 4.3% with respect to AdipoRon, and to 5.5% and 3.2% against Paclitaxel in H1299 and A549 cells, respectively (Figure 5a–d).

Fragmentation of the nuclear DNA is also considered one of the biochemical hallmarks of apoptotic cell death [32]. Conversely, Paclitaxel-induced cytotoxicity has been linked to many forms of cell death in cancers, including the programmed ones [7,33]. Therefore, we screened the potential involvement of apoptosis in the Combo-mediated outcomes by examining the expression levels of both cleaved caspase-3 and PARP. The obtained results revealed a clear activation of both caspase 3 and PARP in response to Combo at all time points tested (Figure 5e,h). As expected, Paclitaxel triggered the cleavage of caspase 3 and PARP as well, especially at 48 h (Figure 5e–j). Impressively, Combo provided a stronger activation of both apoptotic markers at 24 h for H1299 (Figure 5h–j), and at 24 and 48 h in A549 cells (Figure 5e–g).

Overall, these results suggest that AdipoRon enhances Paclitaxel-mediated cytotoxicity mostly by forcing NSCLC cells to undergo apoptosis-mediated cell death induction.

### 3.5. Combination of AdipoRon Plus Paclitaxel Induces AMPK Activation in NSCLC Cells

**Figure 6:**
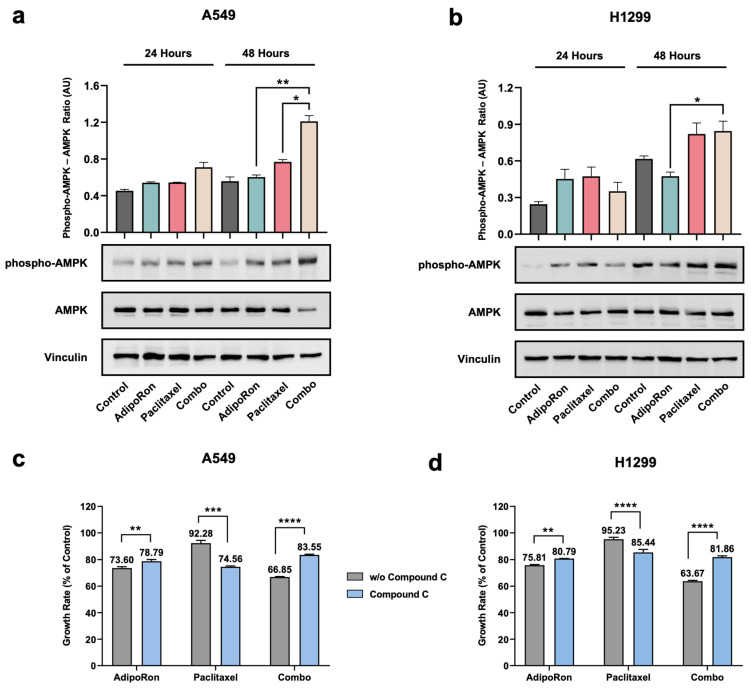
Determination of AMPK involvement in the AdipoRon, Paclitaxel, and Combo-mediated effects in NSCLC cell lines. H1299 and A549 were treated without (Control) or with 10 µg/mL AdipoRon, 4 nM Paclitaxel, and Combo. Thereafter, the activation status of AMPK was addressed at 24 and 48 h. Typical Western blotting images reporting the phospho-AMPK (Thr172), total AMPK, and Vinculin levels in A549 (**a**) and H1299 (**b**), along with the underlying analysis. (**c**) A549 and (**d**) H1299 cells were treated without (Control) or with 10 µg/mL AdipoRon, 4 nM Paclitaxel, and Combo for 24 h, either in the presence or absence of 5 µM of Compound-C. Through direct cell number counting, proliferation index was assessed and reported on bar charts as a proportion of controls. Quantitative data analysis was reported in Arbitrary Units (AU). * *p* < 0.05, ** *p* < 0.01, *** *p* < 0.001, **** *p* < 0.0001 by Brown–Forsythe and Welch ANOVA tests.

Originally stated as a tumor suppressor gene, AMP-activated protein kinase (AMPK) has been shown to either restrain or promote cancer progression, depending on cell type and state [34]. We previously reinforced the essential role of AMPK in mediating the AdipoRon-induced anticancer effects, explicitly in NSCLC cells [21]. Analogously, Paclitaxel-mediated perturbation in the phosphorylation status of AMPK has also been observed in different cancers, including NSCLC [35,36,37]. Therefore, we initially inspected AMPK as one of the possible kinases involved in the Combo outcomes. Single and combined treatments with 10 µg/mL AdipoRon and 4 nM Paclitaxel were thus administered to A549 and H1299 cells for up to 48 h. With respect to untreated cells, a pronounced AMPK phosphorylation was observed in response to AdipoRon and Paclitaxel, either individually or collectively (Figure 6a,b). Intriguingly, we observed that Combo triggered a more robust activation of AMPK than either AdipoRon or Paclitaxel in A549 cells, particularly at 48 h (Figure 6a). Combo-mediated AMPK phosphorylation was similar or slightly greater than that of single agents in H1299 cells instead (Figure 6b).

To corroborate the AMPK involvement in the Combo-mediated cell growth inhibition, we also tested the consequences of the ATP-competitive AMPK inhibitor Compound-C on NSCLC proliferation. Therefore, single and combined treatments were administered to A549 and H1299 cells, either with or without 5 µM Compound-C for 24 h. Fascinatingly, cell growth inspection revealed a dualistic Compound-C outcome in relation to AdipoRon and Paclitaxel single treatment. Whilst the simultaneous presence of Compound-C partially reduced the AdipoRon efficacy in NSCLC cells, its combination with Paclitaxel enhanced the Paclitaxel-mediated anticancer effects (Figure 6c,d). Even more exciting was the cell growth rescue detected in Combo when NSCLC cells were pre-medicated with Compound-C. Precisely, the inhibition rate moved from 33% to 16% in A549 (Figure 6c) and from 36% to 19% in H1299 cells (Figure 6d).

Collectively, these findings suggest that a combination of AdipoRon plus Paclitaxel stimulates AMPK phosphorylation, which is required to promote NSCLC-mediated cell growth inhibition.

### 3.6. Combination of AdipoRon Plus Paclitaxel Downregulates ERK1/2 in NSCLC Cells

**Figure 7:**
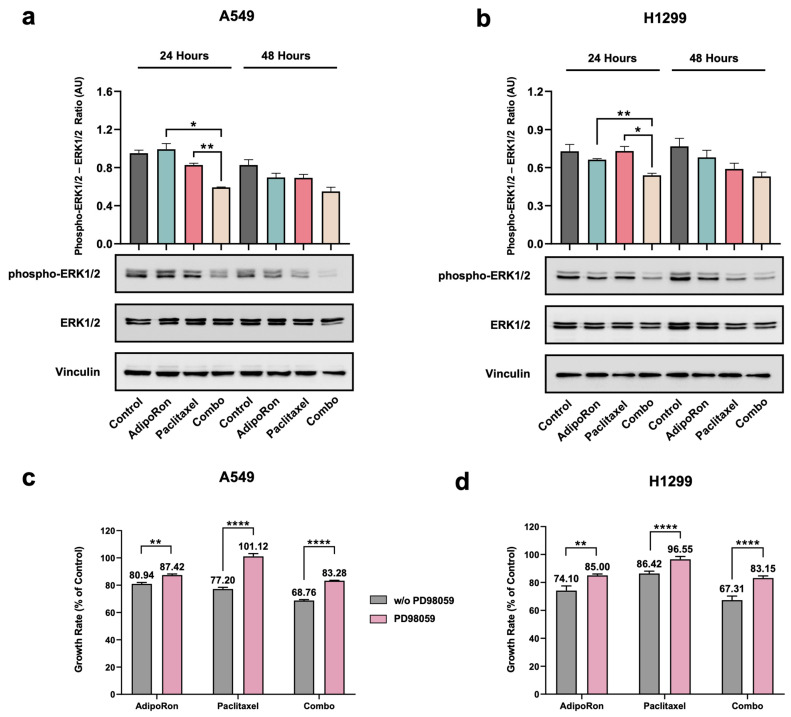
Valuation of ERK1/2 involvement in the AdipoRon, Paclitaxel, and Combo-mediated effects in NSCLC cell lines. H1299 and A549 were treated without (Control) or with 10 µg/mL AdipoRon, 4 nM Paclitaxel, and Combo. Thereafter, the activation status of ERK1/2 was addressed at both 24 and 48 h. Typical Western blotting images reporting the phospho-ERK1/2 (Thr202/Tyr204), total ERK1/2, and Vinculin levels in A549 (**a**) and H1299 (**b**), along with the underlying analysis. (**c**) A549 and (**d**) H1299 cells were treated without (Control) or with AdipoRon (10 µg/mL), Paclitaxel (4 nM), and Combo for 24 h, either in the presence or absence of 10 µM of PD98059. Through direct cell number counting, the proliferation index was assessed and reported on bar charts as a proportion of controls. Quantitative data analysis was reported in Arbitrary Units (AU). * *p* < 0.05, ** *p* < 0.01, **** *p* < 0.0001 by Brown–Forsythe and Welch ANOVA tests.

Extracellular signal–regulated kinase (ERK) 1/2 is another pivotal messenger affecting both proliferation and survival of cancer cells [38,39]. Recently, ERK1/2 activation has been reported as a result of the AdipoRon stimulation in tumor models [13,17,20]. Meanwhile, there is no shortage of evidence supporting the ERK1/2 involvement in the Paclitaxel-related anticancer features [40,41,42]. We therefore inspected the ERK1/2 stimulation in an attempt to provide additional mechanistic details about the combinatorial effects of AdipoRon and Paclitaxel. NSCLC cells underwent pharmacological treatment with 10 µg/mL AdipoRon and 4 nM Paclitaxel for up to 48 h, either individually or together. Irrespective of single-drug administration, the combination of AdipoRon plus Paclitaxel gradually decreased the ERK1/2 phosphorylation over time, both in A549 and H1299 (Figure 7a,b).

To further investigate the ERK1/2 engagement in Combo-mediated effects, we subsequently tested the impact of the MEK1/MEK2 inhibitor PD98059 in our experimental setting. Notably, the usage of 10 μM PD98059 hindered the antiproliferative properties of both single and combined treatments with AdipoRon and Paclitaxel (Figure 7c,d). Almost a total rescue was observed in response to Paclitaxel, while the Combo’s ability to inhibit NSCLC cell growth dropped to half in the presence of PD98059 (Figure 7c,d).

On the whole, these results denote that the combination of AdipoRon plus Paclitaxel affects ERK1/2 phosphorylation, which also participates in the Combo outcome in NSCLC cells.

## 4. Discussion

NSCLC is a highly heterogeneous group of tumors characterized by multiple molecular alterations affecting essential cellular pathways. Chemotherapy and immunotherapy, administered either concurrently or sequentially, represent the current standard of care for NSCLC patients without targetable genetic alterations [3]. Paclitaxel is one of the leading chemotherapeutic agents available against NSCLC [6]. Despite being effective, three main barriers limit the Paclitaxel usage in clinical practice, namely metastasis, chemoresistance, and dose-limiting toxicity [5,6]. Therefore, there is a substantial unmet clinical need to explore novel therapeutic options for curing NSCLC.

In our previous study, we proposed the synthetic adiponectin receptor agonist AdipoRon as an effective anticancer compound in NSCLC [21]. Specifically, AdipoRon impaired viability, proliferation, and clonogenic potential in two cellular models of NSCLC. An adaptive glycolytic dependence was also observed as a result of the AdipoRon administration within these cells. To test the hypothesis that AdipoRon could enhance the Paclitaxel-mediated anticancer effects, we performed an interventional study by combining these two drugs in A549 and H1299 NSCLC cells.

The results achieved in this study clearly support the therapeutic benefits of administering AdipoRon with Paclitaxel in NSCLC. Majorly, cell growth and clonogenic potential were used to address the biological outcomes of both individual and combined treatments. Integrating AdipoRon with Paclitaxel elicited a stronger antineoplastic behavior than either medicine alone in NSCLC cells. Moreover, the assessment of the pharmacokinetic interaction unveiled a synergistic cooperation between AdipoRon and Paclitaxel. Combination therapy is a long-standing pharmacological approach for treating malignant disorders [22]. Synergism represents the most striking kind of combination therapy, as it exhibits greater health benefits than linear cumulative effects. Intriguingly, most of the approved drug combinations in oncology provide additive rather than synergistic efficacy [43]. Conversely, our results designate the combination of AdipoRon plus Paclitaxel as an effective and synergistic treatment to hamper the unbridled NSCLC growth at the preclinical stage. Because Paclitaxel is already a broadly used chemotherapeutic agent, cost and time for subsequent combination trials can be reduced, thereby benefiting the “medically underserved population”.

To investigate the underlying mechanisms, individual and combined treatments were also inspected for changes in cell cycle progression. Remarkably, simultaneous administration of AdipoRon plus Paclitaxel exhibited a stronger cell growth inhibition along with a significant increase in the Paclitaxel-mediated subG1 occurrence. Considering the DNA fragmentation as one of the biochemical hallmarks of apoptosis, we also investigated the contribution of this kind of death after Combo administration. The cleavage abundance of both PARP and Caspase-3 was stronger in Combo than Paclitaxel, supporting a dynamic involvement of the apoptosis-mediated cytotoxicity. As a well-known death inducer, Paclitaxel drives cancer cells to self-annihilation by activating PARP and Caspase-3 [44,45,46]. Moreover, enhancing the Paclitaxel-mediated apoptosis is one of the main targets of employing a combination approach in oncology [47,48]. Nevertheless, Paclitaxel can also trigger other mechanisms of death in malignant cells, such as mitotic catastrophe and autophagy [7,33]. Alternative programmed cell death, like necroptosis and pyroptosis, has recently been reported in response to Paclitaxel [49]. Therefore, the involvement of other cell death mechanisms cannot be neglected in the Combo-mediated outcomes.

Changes in phase-by-phase progression were also unveiled in NSCLC cells receiving a concomitant regimen of AdipoRon and Paclitaxel. With respect to either Paclitaxel or AdipoRon, the percentage of G0/G1 phase appeared markedly reduced in Combo-treated NSCLC cells. Consistently, the number of cells moving toward the G2/M phase was very minimal after the dual stimulation. The literature has amply demonstrated that treating cancer cells with AdipoRon leads to a significant G0/G1 increase, followed by a concomitant reduction in both the S and G2/M phases [12,13,17,20]. In accordance with these findings, we corroborated the AdipoRon feature in restraining cell cycle progression even in NSCLC. The same cannot be claimed for Paclitaxel since microtubular stabilization could lead to a mitotic blockage in the late G2/M phase [50]. Negligible fluctuations were detected in the G2/M amount after Paclitaxel treatments in H1299, while even a slight decrease was recorded in A549 cells. To figure out this data inconsistency, some perspectives must be taken into account: (i) with respect to the employed doses (2 and 4 nM), higher Paclitaxel amounts are usually required to persuade a full blockage in the G2/M phase [7,51,52,53]; (ii) G2/M independent modulations have been observed in the cell cycle at low Paclitaxel dosages [54]; (iii) Paclitaxel-induced apoptosis might also occur independently of a prior G2/M phase arrest [55]. To note, we have already tested these Paclitaxel concentrations in NSCLC cells before, and no G2/M accumulation was detected even at that time [27].

Besides improving efficacy, combination therapy should be aimed at restraining the single-drug toxicity, maybe by reducing the working concentrations. Adverse side reactions constitute one of the limiting steps for Paclitaxel use in clinical practice [6]. Conversely, the proposed Paclitaxel partner (AdipoRon) has shown very low or no toxicity at the preclinical stage [56,57]. Therefore, the choice of using a low Paclitaxel amount in combination with AdipoRon is intended to obtain both enhanced responsiveness and reduced toxicity. To corroborate this assumption, we also tested the Combo outcome in human keratinocyte HACAT cells. As the most common type of epidermal cells, skin may undergo mild to moderate adverse events following Paclitaxel administration [58]. Notably, almost no toxicity was observed after both single and double treatments involving Paclitaxel in these cells. However, a more thorough assessment of the combination’s harmfulness is needed in HACAT cells, as well as in other non-cancer models.

Concerning the signaling pathways, the assessment of AMPK activation substantiated opposite yet crucial roles in single-drug responses. Although both compounds phosphorylated AMPK, the employment of Compound-C constrained and enhanced the antiproliferative effects of AdipoRon and Paclitaxel, respectively. These findings are broadly consistent with existing theories in the oncology field. AdipoRon-mediated AMPK activation has been extensively reported in cancer models [12,13,26]. More recently, we reinforced the essential role of AMPK in the AdipoRon-mediated outcome, demonstrating how its ablation counteracted the effectiveness of AdipoRon in NSCLC cells [21]. Perturbation in AMPK status has largely been connected to Paclitaxel-related survival mechanisms, since its downregulation facilitated growth inhibition and apoptosis occurrence induced by this microtubule-stabilizing agent [36,37,59,60]. Intriguingly, the combination of AdipoRon plus Paclitaxel triggered a powerful phosphorylation of AMPK, while premedication with Compound-C implicated a significant cell growth rescue. Along with the mere activation, the AMPK results emphasize the relevance of other signaling features in influencing the cellular functions. Both individual and combined treatments caused AMPK activation in this study, but not always, as preventing its phosphorylation provided the same outcome. Therefore, future experiments should be aimed at characterizing the involvement of AMPK activation, focusing on signaling duration, spatial and cellular localization, for instance.

ERK1/2 is another pivotal signaling molecule involved in both cancer growth and resistance [38,39]. Our findings elucidated the necessity of ERK1/2 for the Paclitaxel-mediated action, as its inhibition by PD98059 induced a significant rescue in NSCLC cells. ERK1/2 has displayed conflicting evidence with respect to Paclitaxel in cancer models. While preventing Paclitaxel-induced ERK1/2 phosphorylation has been reported to enhance its antineoplastic effects [61,62], rescue outcomes have also been observed in cancer models [63]. With respect to NSCLC, the non-ATP competitive and selective MEK1 and MEK2 inhibitor U0126 has been proven to attenuate Paclitaxel-induced apoptosis [64]. Therefore, it is conceivable that a differential inhibition of the downstream ERK1/2 substrates, as well as varying effects of MEK inhibitors on other signaling molecules, may affect the Paclitaxel-related features in an opposite way. A partial rescue was observed in response to AdipoRon, either alone or in combination with Paclitaxel. At first glance, the results achieved with PD98059 might be difficult to interpret due to the ERK1/2 downregulation obtained after Combo treatments. However, a short-term ERK1/2 activation preceding the feedback suppression could easily explain the outcomes after MEK inhibition via PD98059 incorporation. In this regard, kinetic experiments could elucidate the ERK1/2 involvement in the Combo-mediated effects.

Defining the crosstalk between AMPK and ERK1/2 could be helpful to recapitulate the exact network underlying the Combo-mediated effects in NSCLC. Several studies have disclosed a profound and complicated interplay involving these two pathways [65]. AMPK can act as a downstream effector of MAPK, but at the same time, it regulates MAPK in reverse. With respect to ERK1/2, an inverse correlation with the degree of AMPK phosphorylation has been observed as a result of either glucose deprivation or metformin administration in human acute myeloid leukemia cells [66]. Similarly, AMPK activation can hamper the dual interaction involving eukaryotic elongation factor 2 kinase (eEF2K) and mitogen-activated protein kinase kinase-MAPKK (MEK1/2), disrupting the signaling loop that activates ERK1/2 [67]. The phosphatidylinositol 3-kinase/protein kinase B (PI3K/AKT) pathway also shares a feedback loop with both AMPK and ERK1/2 [68,69]. More generally, future efforts should be aimed at characterizing the engagement of other effectors triggered by Combo in NSCLC. Both computational and proteomic analysis could be useful to achieve this goal [70].

There are also some unanswered questions that confer specific limitations to this study. Firstly, the entire study has been carried out in *in vitro* models; therefore, confirming these results in a more complex biological system, such as *in vivo* methodologies, might endorse the translation of our findings in the subsequent phase of the clinical trials. Secondly, although the combination displayed greater effectiveness in preventing cell growth than Paclitaxel alone, no statistics have been provided regarding its impact on both tumor reoccurrence and resistance. Lastly, careful consideration needs to be given to the genetic fingerprint of the employed NSCLC cells. Although purely coincidental, both A549 and H1299 display a wild-type status for the epidermal growth factor receptor (EGFR). In view of the prevalence and clinical significance of EGFR in NSCLC, our results leave a knowledge gap regarding the responsiveness of EGFR mutants to the combination of AdipoRon plus Paclitaxel.

## 5. Conclusions

In this study, we proved that a combination of AdipoRon and Paclitaxel synergistically enhances cell growth inhibition and impairs colony formation in NSCLC cells. Paclitaxel-mediated cytotoxicity was also potentiated by AdipoRon, since a larger apoptosis induction was observed in Combo than in Paclitaxel. Intriguingly, AMPK and ERK1/2 displayed an active involvement in Combo efficacy, as their inhibition rescued the antiproliferative effects in NSCLC cells.

Overall, a combination of AdipoRon with Paclitaxel provides therapeutic benefits that could be useful to overcome the Paclitaxel resistance in NSCLC. However, future studies are needed to corroborate the efficacy, safety, and underlying mechanisms of this combination in NSCLC therapy.

## Data Availability

The datasets used and/or analyzed during the current study are available from the corresponding author on reasonable request.

## Supplementary Materials

The following supporting information can be downloaded at: https://www.mdpi.com/article/10.3390/cells14080602/s1, Table S1: Cell viability outcomes of AdipoRon, Paclitaxel and the combination of AdipoRon plus Paclitaxel in A549 cells; Table S2: Cell viability outcomes of AdipoRon, Paclitaxel and the combination of AdipoRon plus Paclitaxel in H1299 cells; Figure S1: Investigation of low dosages of AdipoRon plus Paclitaxel in H1299 cells.

## Abbreviations

The following abbreviations are used in this manuscript:

NSCLC	Non-Small Cell Lung Cancer
AMPK	AMP-activated protein Kinase
ERK1/2	Extracellular Signal-Regulated Kinase 1/2
FDA	Food and Drug Administration
PDAC	Pancreatic Ductal Adenocarcinoma
MAPK	Mitogen-Activated Protein Kinases
mTOR	Mammalian Target Of Rapamycin
NF-κB	Nuclear Factor Kappa-light-chain-enhancer of activated B cells
RIPKs	Receptor-Interacting Protein Kinase Family Members
ULK1	Unc-51-Like autophagy-activating Kinase
PI	Propidium iodide
CFA	Colony Formation Assay
HAS	Highest Single Agent
PARP	Poly ADP Ribose Polymerase
MEK1/2	Mitogen-Activated Protein Kinase Kinase-MAPKK
PI3K	Phosphatidylinositol 3-Kinase
AKT	Protein Kinase B
