# Supplementary material for: Combining AdipoRon with Paclitaxel Unveils Synergistic Potential in Non-Small Cell Lung Cancer Cells via AMPK-ERK1/2 Signaling
Source: Cells. 2025 Apr 16;14(8):602. doi: 10.3390/cells14080602 (PMC12026066; doi:10.3390/cells14080602)
Supplement: Supplementary file 1 [file cells-14-00602-s001.zip › cells-3538620-supplementary.pdf]

Supplementary Table S1: Cell viability outcomes of AdipoRon, Paclitaxel and the combination of AdipoRon plus Paclitaxel in A549 cells.

Treatments	% Viability (Mean ± SD)
Control	100 ± 7.66
AdipoRon 2.5 ug/mL	79.74 ± 6.13
AdipoRon 5 ug/mL	65.20 ± 6.24
AdipoRon10 ug/mL	53.69 ± 4.77
Paclitaxel 1 nM	87.90 ± 6.42
Paclitaxel 2 nM	72.93 ± 5.70
Paclitaxel 4 nM	46.37 ± 4.41
Combo 2.5+1	71.03 ± 1.94
Combo 2.5+2	54.16 ± 3.10
Combo 2.5+4	41.45 ± 3.79
Combo 5+1	53.43 ± 2.22
Combo 5+2	45.23 ± 2.06
Combo 5+4	40.45 ± 2.50
Combo 10+1	36.95 ± 2.43
Combo 10+2	36.22 ± 0.65
Combo 10+4	33.22 ± 2.66

Supplementary Table 2: Cell viability outcomes of AdipoRon, Paclitaxel and the combination of AdipoRon plus Paclitaxel in H1299 cells.

Treatments	% Viability (Mean ± SD)
Control	100 ± 4.82
AdipoRon 5 ug/mL	101.08 ± 3.87
AdipoRon 10 ug/mL	85.03 ± 3.40
AdipoRon 20 ug/mL	78.55 ± 3.22
Paclitaxel 2 nM	94.97 ± 6.27
Paclitaxel 4 nM	87.18 ± 3.66
Paclitaxel 8 nM	81.03 ± 5.17
Combo 5+2	79.69 ± 7.37
Combo 5+4	82.35 ± 3.65
Combo 5+8	73.99 ± 4.05
Combo 10+2	73.24 ± 6.18
Combo 10+4	72.94 ± 5.22
Combo 10+8	74.61 ± 5.28
Combo 20+2	83.21 ± 4.24
Combo 20+4	78.22 ± 1.09
Combo 20+8	64.15 ± 2.67

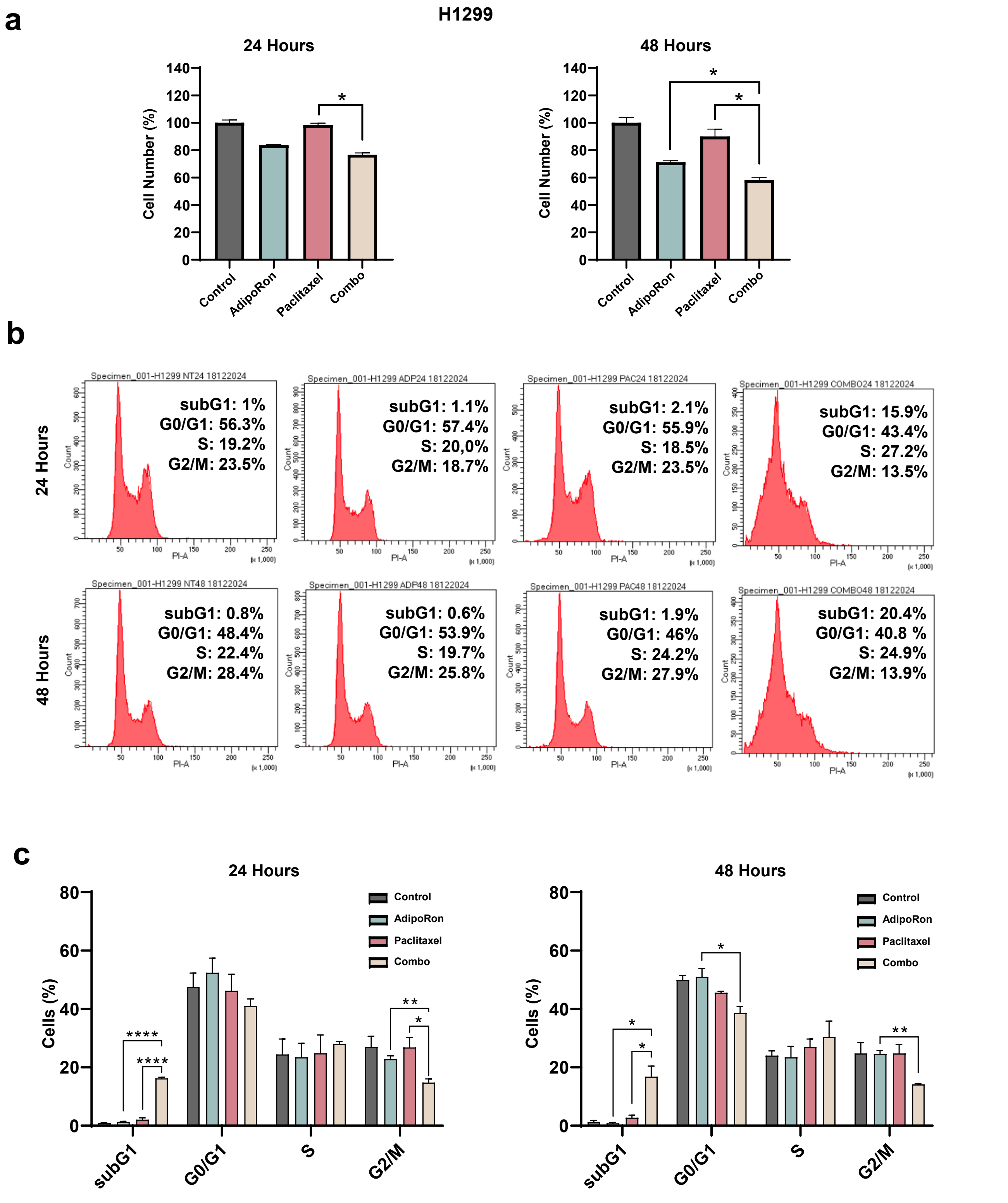

Supplementary Figure S1. *Investigation of the low dosages of AdipoRon plus Paclitaxel in H1299 cells.* (**a**) Growth response rate achieved in presence of 5 µg/mL AdipoRon, 2 nM Paclitaxel, and their combination (Combo) at 24 and 48 hours. (**b**) Representative cell cycle profile obtained in the same experimental setting at 24 and 48 hours, respectively. (**c**) Statistical analysis of multiple cell cycle experiments carried out at 24 and 48 hours. Data is represented in percentage as Mean ± Standard deviation. *p < .05, **p < .01, ***p < .001, ****p < .0001 by Brown-Forsythe and Welch ANOVA tests.
